# Expression and clinical significance of the p53/SAT1/ALOX15 ferroptosis‐associated proteins in sinonasal inverted papilloma

**DOI:** 10.1002/wjo2.213

**Published:** 2024-09-04

**Authors:** Dan‐Yang Li, Lin Wang, Ji‐Sheng Zhang, Jia‐Jia Zi, Han Chen, Zi‐Hui Dong, Long‐Gang Yu, Yan Jiang

**Affiliations:** ^1^ Department of Otorhinolaryngology Head and Neck Surgery The Affiliated Hospital of Qingdao University Qingdao China

**Keywords:** ferroptosis, p53, pathogenesis, sinonasal inverted papilloma

## Abstract

**Objectives:**

Sinonasal inverted papilloma (SNIP) is a benign tumor type that has been subject to growing levels of research interest owing to its potential for malignant transformation. However, there have been no studies to date of ferroptosis or related proteins in SNIP. Accordingly, this study was designed to examine correlative relationships between SNIP pathogenesis and the expression of proteins associated with ferroptotic activity, including p53, SAT1, and ALOX15.

**Methods:**

Samples were collected from 44 total SNIP patients, and control middle turbinate samples were obtained from 28 patients with deviated septums. The RNA and protein levels of p53, SAT1, and ALOX15 were compared between these samples via quantitative real‐time PCR (qRT‐PCR), Western blot analysis (WB), and immunohistochemistry (IHC). The expression of mRNA was further validated by interrogating the GSE193016 data set. The correlations among the expression levels of these three genes were also assessed. Then, the Krouse stage system was used to grade these patients and to explore differences in p53, SAT1, and ALOX15 expression among different stages of the disease. Lastly, we compared the differences in the expression of these genes in inverted papilloma and squamous cell carcinoma by qRT‐PCR and IHC.

**Results:**

SNIP samples exhibited significantly higher p53, SAT1, and ALOX15 mRNA and protein levels than control samples, and strong correlations were observed between the levels of these three proteins. Furthermore, the expression levels of p53, SAT1, and ALOX15 were significantly higher in stage T4 compared to T2 in SNIP. p53 and SAT1 were significantly elevated in squamous carcinomas compared to inverted papilloma. However, the expression of ALOX15 tended to decrease in squamous carcinoma.

**Conclusion:**

These results support a potential role for the p53/SAT1/ALOX15 ferroptotic pathway proteins in SNIP pathogenesis, although future molecular biology‐based studies will be essential to test this hypothesis.

## INTRODUCTION

Sinonasal papillomas are benign tumors derived from the sinuses and nasal cavities that exhibit a high risk of recurrence.^1^ These lesions are generally classified into Schneiderian or inverted papilloma (SNIP), exophytic papilloma (EP), and oncocytic papilloma (OP) cases,^2^ among which SNIP lesions are the most common.^3^ Even so, SNIP lesions comprise just 0.5%–7% of all nasal sinus tumors, affecting 0.2–1.5 per 100,000 persons annually.^4^ SNIP generally presents with symptoms including unilateral nasal congestion, sinus infection with nasal discharge, and rhinorrhea. Bowing to their high rates of locally invasive growth and recurrence, they also have the potential to undergo malignant transformation into squamous cell carcinoma (SCC).^4^, ^5^, ^6^ Research focused on SNIP pathogenesis has, to date, been relatively limited, with these lesions being believed to be attributable to chronic inflammation, environmental factors such as exposure to organic solvents, and infection with human papillomavirus.^2^, ^4^, ^7^, ^8^ Inverted papilloma (IP) often exhibits pronounced increases in the level of infiltration by T cells, macrophages, and neutrophils consistent with their status as immunologically active lesions.^9^

Cell death is integral to appropriate organismal growth and development through its ability to help maintain homeostasis, although it is also closely tied to the incidence of many forms of disease. Studies in recent years have highlighted the importance of non‐apoptotic programmed cell death pathways while also elucidating the molecular processes that underlie them.^10^ It has been indicated that the apoptosis index is significantly elevated in IP cases with mild and moderate dysplasia compared with IP with carcinoma, invasive SCC, and EP. Our previous study reported that the autophagy‐related genes LC3B, ATG5, and Beclin1, as well as HMGB1, were significantly expressed in SNIP.^11^, ^12^ However, there is still a lack of research on the specific mechanisms and potential modes of apoptosis involved in SNIP.

Ferroptosis is one such relatively recently identified mode of cell death characterized by the accumulation of toxic levels of lipid hydroperoxides in an iron‐dependent manner, resulting in morphological characteristics including greater membrane density, the loss of mitochondrial cristae, and marked mitochondrial atrophy.^13^, ^14^, ^15^ The p53/SAT1/ALOX15 pathway is central to regulating ferroptotic activity, with SAT1 serving as a p53 metabolic target that subsequently enhances the expression of ALOX15 to drive reactive oxygen species (ROS) biogenesis and ferroptosis.^16^

Ferroptosis has been tied to various head and neck diseases, including head and neck squamous carcinoma wherein IL‐6 drives tumor progression via the induction of ferroptosis resistance while inducing ferroptotic death in head and neck cancer cells can overcome resistance to cisplatin.^17^, ^18^ The precise association between ferroptosis and SNIP development, however, remains uncertain. Here, the expression of the ferroptosis‐related p53, SAT1, and ALOX15 genes was assessed in SNIP patient tissues in an effort to better ascertain the relevance of ferroptosis to SNIP pathogenesis.

## MATERIALS AND METHODS

### Patient samples

This study utilized samples of tissue collected from 44 SNIP patients hospitalized from 2020 to 2023, all of whom underwent nasal endoscopic surgery and had pathologically confirmed SNIP diagnoses. SNIP staging in these patients was performed as the Krouse classification system.^19^ As a control, we obtained nasal mucosal tissues from the middle turbinate of 20 patients with only septal deviation. Besides, tumor tissues from 10 patients with squamous cell carcinoma (SNSCC) without a previous history of SNIP were collected. Patient clinical and demographic characteristics are presented in Table 1. All patients provided informed consent, and the Affiliated Hospital of Qingdao University provided ethical approval for this study (QYFY WZLL 28236).

**Table 1:** Patients' demographics and clinical characteristics.

Variables	Control (*n* = 20)	SNIP (*n* = 44)	SNSCC (*n* = 10)
Gender (male), *n* (%)	13 (65.0)	28 (63.6)	6 (60.0)
Age (years)^a^	46.0 (42.0, 57.0)	54.5 (49.0, 66.0)	55.0 (45.0, 62.5)
Krouse T stage, *n* (%)			
T1	‐	‐	‐
T2	‐	12 (27.2)	‐
T3	‐	16 (36.4)	‐
T4	‐	16 (36.4)	‐

Abbreviations: SNIP, Sinonasal inverted papilloma; SNSCC, sinonasal squamous cell carcinoma.

^a^ Data are presented as median (Q1, Q3).

### RNA isolation and quantitative real‐time PCR (qRT‐PCR)

Total RNA in the tissues was obtained by extraction using Trizol (Vazyme Company). The RNA was then converted to cDNA by HiScript II Q RT SuperMix for qPCR (Vazyme Company), and the qPCR reaction was performed with ChamQ SYBR qPCR Master Mix (Vazyme Company). The relative gene expression was calculated by the 2‐ΔΔCt formula. The primers used for qRT‐PCR are described in Table S1.

### Western blot (WB) analysis

Total proteins were extracted using RIPA solution (Solarbio) and quantified using a BCA protein assay kit (Elabscience). Subsequently, equal amounts of proteins were electrophoresed on Tris‐Tricine SDS‐PAGE and transferred onto polyvinylidene difluoride (PVDF) membranes. The antibodies employed in this study included α‐Tubulin (NP_001265481) (ABclonal), p53 (NP_000537) (abmart), SAT1 (NP_002961) (Bioss), and ALOX15 (NP_001131) (Bioss). Following that, the membranes were incubated with corresponding secondary antibodies (Elabscience) for 1 h, and the signal was detected using an ECL chemiluminescent ultrasensitive kit (Meilun). The visualized bands were captured using an Alliance Q9 imaging scanner (UVITEC), and the band intensities were quantified using ImageJ 6.0 (Media Cybernetics, Inc.).

### Immunohistochemistry (IHC) staining

Briefly, we deparaffinized and rehydrated patient tissue sections before blocking endogenous peroxidase with 3% H_2_O_2_ and recovering antigen with 10 mmol/L nitrate buffer. Subsequently, the sections were incubated with 10% goat serum for 1 h at room temperature (RT), followed by incubation with antibodies at 4°C. The sections were then incubated in horseradish peroxidase‐conjugated secondary antibodies and then color developed with DAB solution. The sections were observed, imaged, and analyzed using a microscope and ImageJ software.

### Statistical analysis

Statistical analysis was performed using SPSS 22.0 for data analysis and GraphPad Prism 8.0 for data visualization. If the data followed a normal distribution, the Student's *t*‐test was employed to compare the two groups, and data were expressed as means ± SEM. However, if the data did not conform to a normal distribution, the median and interquartile ranges were used to present the data, while the Mann‐Whitney *U* test was utilized for comparing two groups. Multiple groups were performed using the Kruskal–Wallis test with the Benjamini–Hochberg procedure for multiple hypotheses testing correction. Categorical data were assessed using chi‐square tests or Fisher's exact tests. The Spearman correlation test was used to assess correlations. A significance threshold of *p* < 0.05 was established for all analysis.

## RESULTS

### p53/SAT1/ALOX15 mRNA levels

**Figure 1:**
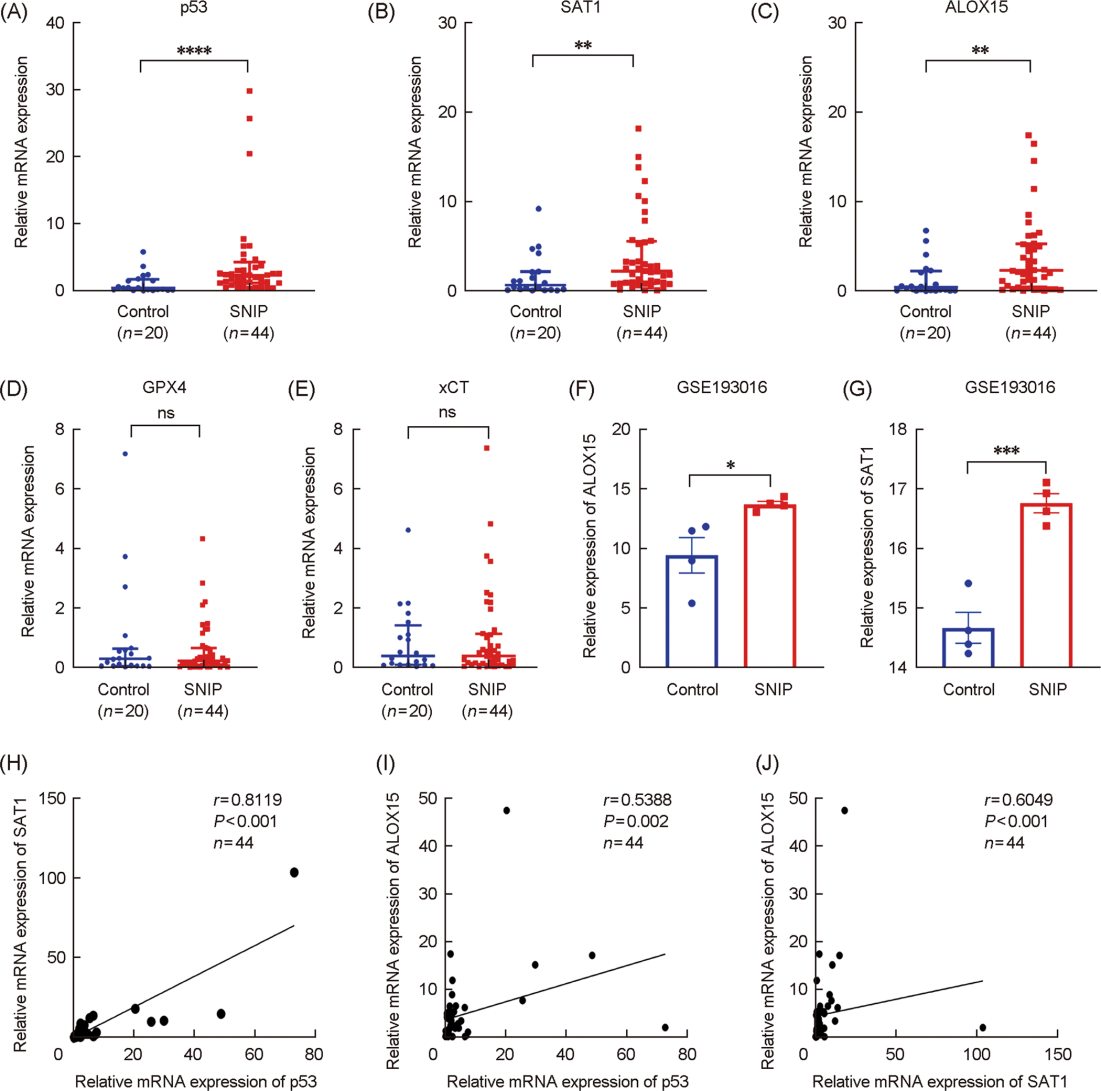
p53/SAT1/ALOX15 mRNA levels. (A–E) The mRNA expression levels of p53, SAT1, ALOX15, GPX4, and xCT in tissues from SNIP (*n* = 44) and control samples (*n* = 20) by qRT‐PCR experiment. (F, G) Relative expression of SAT1 and ALOX15 in GSE193016. (H, I) p53 mRNA expression levels correlated positively with SAT1 and ALOX15 in SNIP. (J) SAT1 mRNA expression levels correlated positively with ALOX15 in SNIP. *p < 0.05; **p < 0.01; ***p < 0.001; ****p < 0.0001; ns, no significance. qRT‐PCR, quantitative real‐time polymerase chain reaction. SNIP, Sinonasal inverted papilloma.

Initially, qRT‐PCR was employed to examine p53, SAT1, and ALOX15 expression while also assessing the classical ferroptosis suppressor genes GPX4 and xCT. As shown in Figure 1A–C, significant overexpression of p53, SAT1, and ALOX15 was evident in SNIP patients, whereas GPX4 and xCT levels were unchanged (Figure 1D,E), such that they were not analyzed further. The upregulation of SAT1 and ALOX15 in SNIP samples was further validated with the GSE193016 transcriptomic data set (Figure 1F,G). Correlation analyses indicated that p53, SAT1, and ALOX15 expression levels were strongly correlated (Figure 1H–J).

### Protein level analyses of p53/SAT1/ALOX15 expression

**Figure 2:**
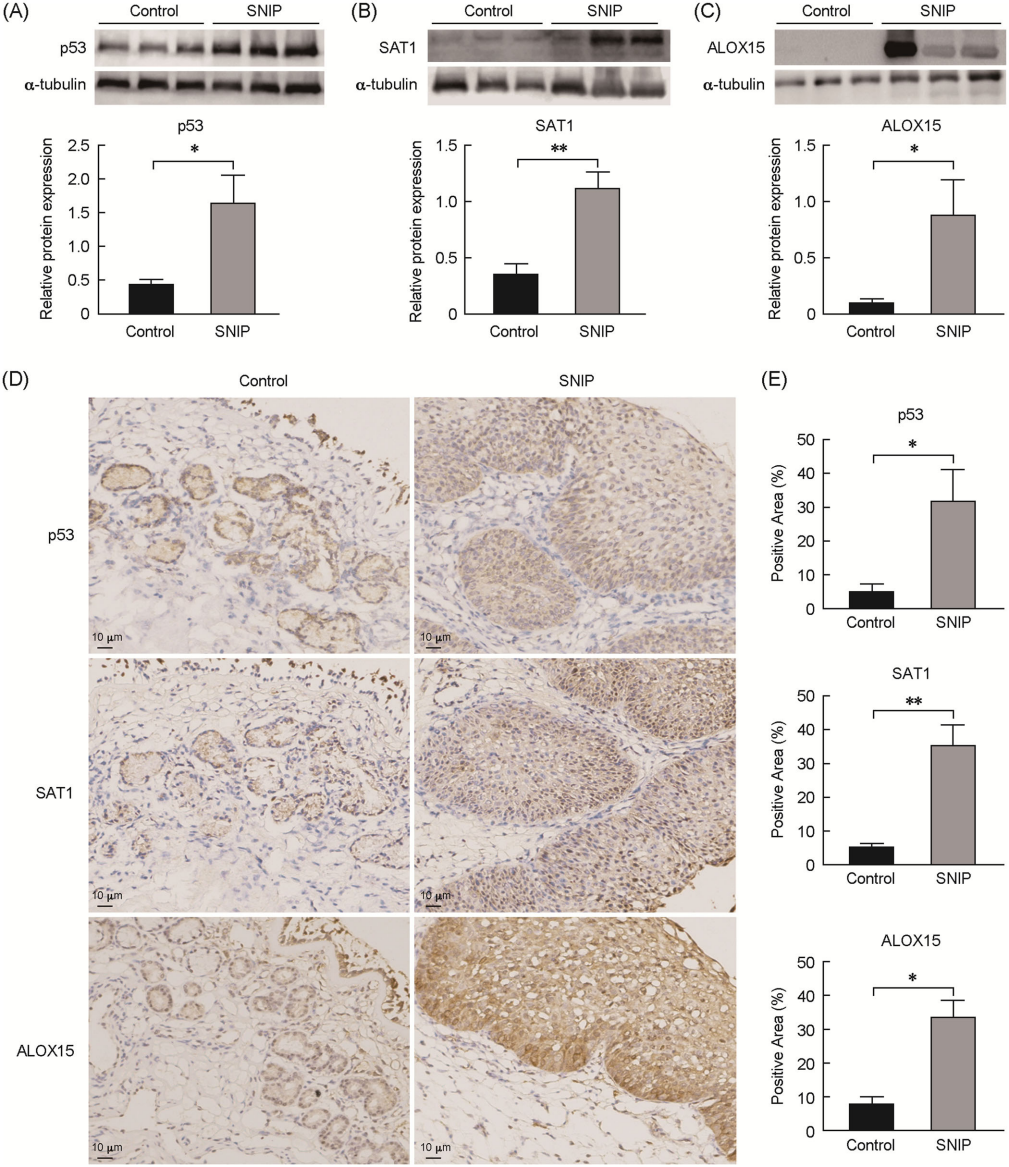
WB and IHC experiments to validate the protein expression of p53/SAT1/ALOX15 in tissues from SNIP and control samples. (A–C) The protein expressions of p53, SAT1, and ALOX15 in SNIP and control patients were examined by WB. Quantification of P53, SAT1, and ALOX15 immunoreactivity in control (*n* = 6) and SNIP patients (*n* = 6) using ImageJ software. (D) IHC for p53, SAT1, and ALOX15 in tissues from SNIP and nasal mucosa from control patients (magnification, 200×). (E) Quantification of p53, SAT1, and ALOX15 immunoreactivity in CRSwNP (*n* = 5) and non‐CRS patients (*n* = 5) using ImageJ software. *p < 0.05, **p < 0.01. IHC, Immunohistochemical. SNIP, sinonasal inverted papilloma; WB, Western blot analysis.

Next, p53, SAT1, and ALOX15 protein levels were further compared between SNIP and control samples via immunohistochemistry and Western blot analysis. The latter of these approaches confirmed a clear rise in p53, SAT1, and ALOX15 levels in SNIP samples (Figure 2A–C), while the former revealed high levels of p53, SAT1, and ALOX15 expression in both the epithelium and the mesenchyme (Figure 2D,E).

### Trends in ferroptotic gene expression as a function of SNIP staging

**Figure 3:**
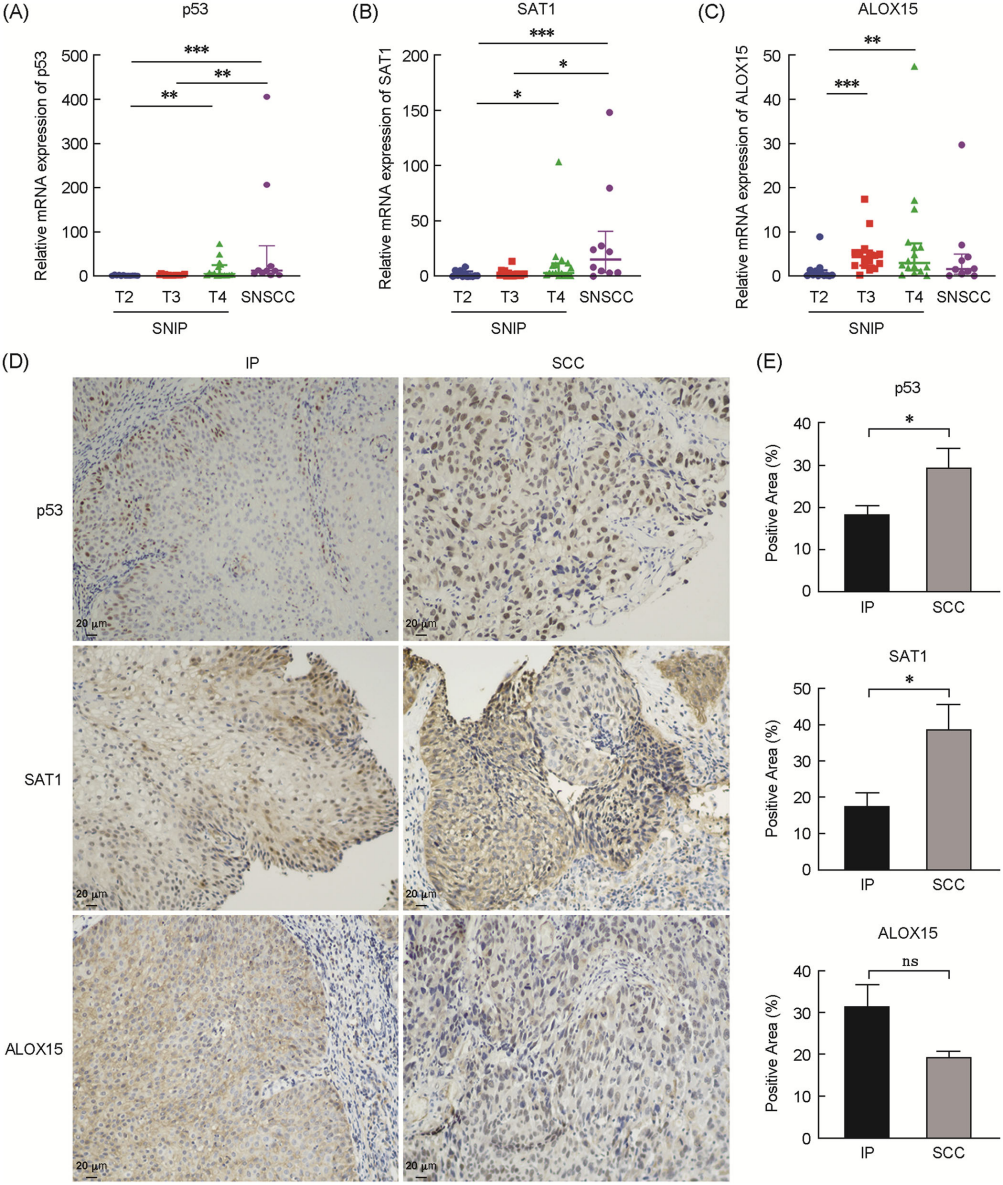
Trends in ferroptotic gene expression of p53/SAT1/ALOX15 as the disease stage progresses. (A–C) The mRNA expression levels of p53, SAT1, and ALOX15 in different study groups were detected by qRT‐PCR experiment. (D) IHC for p53, SAT1, and ALOX15 between the inverted papilloma and squamous cell carcinoma in the same biopsies (magnification, 200×). (E) Quantification of p53, SAT1, and ALOX15 (*n* = 3) immunoreactivity using ImageJ software. *p < 0.05, **p < 0.01, ***p < 0.001; ns, no significance. qRT‐PCR, quantitative real‐time polymerase chain reaction. SNIP, sinonasal inverted papilloma; SNSCC, sinonasal squamous cell carcinoma.

Lastly, differences in p53, SAT1, and ALOX15 levels were compared among SNIP tissues of different stages at the mRNA level. Gradual increases in the expression of p53 and SAT1 were detected with disease progression (Figure 3A,B), including a significant rise from stage T2 to T4 lesions and an upward trend from stage T2 to T3 and stage T3 to T4 lesions. Similarly, increases in ALOX15 expression were observed in stages T3 and T4 compared with stage T2 (Figure 3C). This further supports a potential role for these genes in disease progression.

### Differential expression of p53/SAT1/ALOX15 in SNIP and SNSCC

Next, we further explored the expression changes of p53/SAT1/APOX15 among inverted papilloma and squamous cell carcinoma. First, we selected three patients with pathologic diagnosis of inverted papilloma with squamous cell carcinoma. The same biopsies which included both the inverted papilloma and squamous cell carcinoma showed by IHC that the expression of p53 and SAT1 tended to be elevated in the SCC region, whereas ALOX15 had a decreasing trend (Figure 3D,E). Finally, we further explored the expression of P53/SAT1/ALOX15 by PCR in patients diagnosed with squamous carcinoma only, and the results showed that the expression of P53 and SAT1 in SNSCC had a trend of elevation and ALOX15 had a trend of decreasing on the contrary compared with stage T4 patients in SNIP (Figure 3A–C).

## DISCUSSION

SNIPs are rare benign tumors that have the potential to recur and transform into malignant tumors such that they place a significant financial burden on patients and represent a threat to their lives if not adequately addressed.^4^, ^20^ However, studies of SNIP pathogenesis are, at the molecular level, lacking at present. The pathway of SNIP is receiving increasing attention. It has been demonstrated that the expression of beta‐catenin and cyclin D1 in the WNT pathway is elevated in SNIP and also showed a significant positive correlation with IP grade.^21^ Other studies reported that the activation of AKT and MAPK signaling pathways plays a role in the aggravation of SNIP.^22^, ^23^ No prior studies have documented any role of ferroptosis in the pathogenesis of these lesions. Accordingly, this study was designed to examine the possible links between SNIP and ferroptosis pathway proteins so as to better explain the mechanisms that give rise to these lesions while potentially aiding the formulation of better‐targeted therapies to treat this disease.

Sometimes referred to as the “guardian of the genome”, p53 serves as an essential regulator of cellular survival and division under conditions of stress. In addition to influencing autophagy, cell cycle progression, and apoptosis, p53 can also influence ferroptosis at the transcriptional and posttranslational levels.^24^ Ferroptosis is a form of regulated cell death that is iron dependent and driven by ROS production and characteristic lipid peroxidation.^25^ Spermidine/Spermine N1‐acetyltransferase 1 (SAT1) serves as a key global rate‐limiting polyamine catabolic enzyme, with polyamines being critical for proper growth and the regulation of potassium channels. Under most conditions, SAT1 expression is low, with the upregulation of this gene having been linked to tissue damage in various pathological contexts.^26^, ^27^, ^28^ The lipid peroxidase enzyme lipoxygenase (LOX) has been shown to play roles in many physiological processes and the pathogenesis of inflammatory and hyperproliferative conditions. There are six LOX isoforms encoded in the human genome, including ALOX15.^29^, ^30^ ALOX15 is responsible for catalyzing polyunsaturated fatty acid oxidation, particularly for ω‐6 and ω‐3 fatty acids, yielding biologically active lipid metabolites that are associated with the production of inflammation‐related cytokines.^31^ ALOX15 can reportedly play a key role in the pathogenesis of many diseases including asthma, allergic rhinitis, and cancers of the head and neck.^32^, ^33^

Our study provides the first evidence for differences in SAT1 and ALOX15 expression in SNIP. Specifically, qRT‐PCR, WB, and IHC strategies all confirmed the higher levels of p53, SAT1, and ALOX15 in SNIP lesions, with strong correlations among the expression levels of these three genes, suggesting that SAT1 and ALOX15 may function downstream of p53. Indeed, the p53‐SAT1‐ALOX15 axis has been established as a classical ferroptotic pathway.^13^, ^16^ In SNIP lesions, p53 may undergo specific targeting to drive SAT1 upregulation, in turn influencing the expression of ALOX15. As a result, these processes trigger higher levels of ferroptosis induced by lipid peroxidation.

Previous studies showed that p53 protein accumulates in malignant tumors arising from inverted papilloma.^34^ Comparison of different SNIP tissue stages additionally revealed that p53, SAT1, and ALOX15 expression was much greater in stages T3 and T4 compared with stage T2, suggesting that they may be consistent with the progression of SNIP. These reflect adaptive changes in response to increasing malignant potential and may be related to the cell's attempts to maintain its stability and prevent carcinogenesis. Activation of SAT1 expression induces lipid peroxidation and causes ferroptosis in response to ROS‐induced stress. This mechanism may be an attempt by cells to eliminate potentially cancerous cells by enhancing ferroptosis during the precancerous stage to prevent cancer development.

Then, we further explored the difference between SNIP and SNSCC in this pathway. IHC showed in the same biopsies which included both the inverted papilloma and squamous cell carcinoma, the expression of p53 and SAT1 in SCC was further elevated, while there was a slight decrease in the expression of ALOX15. After that, we collected additional tumor tissues from SNSCC for mRNA detection, and similar to the previous results, P53 and SAT1 were significantly elevated in SNSCC, but the expression level of ALOX15 had a tendency to decrease, in comparison with stages T3 and T4 in SNIP. This demonstrated that ALOX15 is differentially regulated in SNIP and SNSCC. The high levels of p53 and SAT1 may reflect the persistence of DNA damage and oxidative stress within cancer cells, which normally activate the function of p53.^27^, ^35^ However, the decrease of ALOX15 may be an adaptive change adopted by cancer cells to escape ferroptosis. Cancer cells may reduce the occurrence of lipid peroxidation and ferroptosis by decreasing ALOX15 expression, thereby enhancing their own survival. In addition, a variety of cancers including head and neck cancer exhibit a reduction in ALOX15 levels which could be intricately linked to the cancerous microenvironment. Within this environment, there may be certain elements, such as secreted proteins or metabolites, that act to suppress the activity or expression of ALOX15 and then help cancer cells evade ferroptosis. Also, other signaling molecules may influence the regulatory network of ALOX15.^32^, ^36^, ^37^ Further studies will help to reveal the detailed course of these mechanisms and may provide new targets for preventing malignant regression of SNIP.

These results are significant as they underscore the need to further delve into the role of ferroptosis in the pathogenesis of inverted papilloma. This exploration could ultimately enhance our understanding of the disease mechanism. There are several limitations to this study. We need to increase the number of SNIP samples to detect changes in protein levels to ensure the accuracy of our results. Due to the small number of squamous cell carcinoma tissues obtained, we did not further group them to explore the differences in expression of these genes. Most notably, more in‐depth molecular biology studies will be essential to clarify the expression and mechanistic roles of these ferroptosis‐related proteins at the cellular level. Moreover, there is a need for further studies of ferroptotic activity and ROS levels in SNIP tissue samples. Lastly, the predictive utility of these key proteins as tools for disease staging remains to be established.

## CONCLUSION

In summary, the ferroptosis‐related p53, SAT1, and ALOX15 proteins were confirmed to be overexpressed in SNIP lesions, with their levels of expression varying in response to changes in the disease. And these genes may be targets for preventing malignant regression of SNIP. The results offer a foundation for future mechanistic research focused on the clinical relevance of ferroptosis in SNIP.

## AUTHOR CONTRIBUTIONS

Danyang Li, Longgang Yu, Lin Wang, and Jiajia Zi contributed to the conception and design of the study. Lin Wang and Han Chen collected and provided the sample for this study. Danyang Li and Zihui Dong performed data collection and analysis. Danyang Li wrote the first draft of the manuscript; Yan Jiang and Longgang Yu commented on previous versions of the manuscript. All the authors approved the final article.

## CONFLICT OF INTEREST STATEMENT

All authors approved the final manuscript and the submission to this journal. The authors declare that they have no conflicts of interest.

## ETHICS STATEMENT

All patients provided informed consent, and the Affiliated Hospital of Qingdao University provided ethical approval for this study (QYFY WZLL 28236).

## Supporting information

Supporting Information.

## Data Availability

The data are available from the corresponding author on reasonable request.
