# Supplementary material for: Expression and clinical significance of the p53/SAT1/ALOX15 ferroptosis‐associated proteins in sinonasal inverted papilloma
Source: World J Otorhinolaryngol Head Neck Surg. 2024 Sep 4;11(2):281–9. doi: 10.1002/wjo2.213 (PMC12172092; doi:10.1002/wjo2.213)
Supplement: Supplementary file 1 — Supporting Information. [file WJO2-11-281-s001.docx]

Table S1. Primer sequences designed for quantitative real-time polymerase chain reaction (qRT-PCR)

| Gene | ACCESSION | Primer sequence |
| --- | --- | --- |
| P53 | NM_000546 | Forward: 5’- CCTCAGCATCTTATCCGAGTGG-3’ |
|  |  | Reverse: 5’- TGGATGGTGGTACAGTCAGAGC-3’ |
| SAT1 | NM_002970 | Forward: 5’- TACCACTGCCTGGTTGCAGAAG-3’ |
|  |  | Reverse: 5’- CTTGCCAATCCACGGGTCATAG-3’ |
| ALOX15 | NM_001140 | Forward: 5’- ACCTTCCTGCTCGCCTAGTGTT-3’ |
|  |  | Reverse: 5’- GGCTACAGAGAATGACGTTGGC-3’ |
| 18 s rRNA | NR_003286 | Forward: 5’- CTGGATACCGCAGCTAGGAA-3’ |
|  |  | Reverse: 5’- GAATTTCACCTCTAGCGGCG-3’ |
